# Anatomic and Functional Reconstruction of the Abdominal Wall in Prune Belly Syndrome: A Case Report

**DOI:** 10.7759/cureus.75883

**Published:** 2024-12-17

**Authors:** Georgios E Papanikolaou, Georgios Aravanis, Dimitrios N Varvarousis, Aikaterini Kitsouli, Aristidis Tsakiris

**Affiliations:** 1 Plastic Surgery, 401 General Military Hospital of Athens, Athens, GRC; 2 Medical School, University of Ioannina, Ioannina, GRC; 3 Orthopaedics, University General Hospital of Ioannina, Ioannina, GRC

**Keywords:** abdominal fascia plication, abdominoplasty, dynamic abdominal wall reconstruction, muscle transfer, prune belly syndrome

## Abstract

Prune belly syndrome (PBS), or Eagle-Barrett syndrome, is a rare congenital disorder marked by abdominal wall muscle deficiency, urinary tract anomalies, and cryptorchidism, causing significant abdominal wall laxity and functional impairment. This case report discusses an innovative approach to abdominal wall reconstruction in a 19-year-old male patient with PBS and associated conditions, including chronic renal failure and spina bifida. Previously, he underwent distal ureterectomy and vesicoureteral reimplantation at the age of two years to correct urinary tract dilation and bilateral orchiopexy. Preoperative examination revealed a distended abdomen, hypoplastic anterior abdominal wall musculature with associated abdominal flaccidity, and the presence of a malformed umbilical remanent. Due to the extensive abdominal muscle deficiency, the surgical team combined abdominoplasty with bilateral rectus femoris and sartorius muscle transfers. This dynamic reconstruction aimed to enhance abdominal wall function and aesthetics. Postoperative complications included skin flap necrosis, effectively managed with debridement, an abdominal anchor device, and skin grafting. Over a 41-month follow-up, the patient showed marked improvement in abdominal strength, posture, and ambulation, with no recurrence of abdominal bulging. This case highlights the potential of combining fascial plication and muscle transfer for sustained functional and aesthetic benefits in PBS, supporting its consideration in similar complex cases.

## Introduction

Prune belly syndrome (PBS), known also as Eagle-Barrett syndrome, is a rare congenital disorder characterized by the pathognomonic triad of abdominal wall musculature deficiency, cryptorchidism, and urinary tract abnormalities [1]. Abdominal wall laxity and distension confer the characteristic prune-like appearance of the abdomen in those patients. The incidence of PBS is approximately 3.6 to 3.8 per 100,000 live births, with 95% of cases occurring in male patients [2]. Interestingly, these living children have a high frequency of other associated anomalies involving cardiopulmonary (49%), musculoskeletal (65%), and gastrointestinal (63%) systems [3].

The severity of the neonatal presentation of PBS patients determines survival among survivors. About 40% of the newborns are premature, whereas perinatal mortality ranges from 10% to 25%, primarily depending on the grade of pulmonary hypoplasia secondary to oligohydramnios [4]. On the other hand, the prognosis of children with PBS is associated with the degree of urinary tract anomalies and concomitant cardiopulmonary disorders. Particularly, the presence of renal dysplasia, vesicoureteral reflux (VUR), and recurrent episodes of urinary tract infections have an important negative impact on the long-term survival of PBS patients, while favorable prognostic factors are the presence of at least one normal functional kidney and a serum creatinine level less than 0.7 mg/dL during childhood [5,6].

Given the fact that the etiology of PBS remains unknown, with different theories being proposed, the diagnosis during the second trimester through prenatal ultrasound is crucial for early detection and an appropriate intervention [6]. Recently, genetic screening demonstrated that some PBS cases are associated with DNA mutations in known genes that control embryonic genitourinary myogenesis [5].

The severity of abdominal wall flaccidity depends on the presence of complete or partial abdominal wall musculature deficiency [6]. Consequently, abdominal wall reconstruction techniques have the purpose of providing adequate abdominal wall strength and, therefore, long-term cosmetic and functional improvement in adult patients with PBS.

This case report describes a rare and complex approach to dynamic abdominal wall reconstruction in a young adult with PBS. Due to the severe muscle deficiency, the team used an innovative method combining abdominoplasty with rectus femoris and sartorius muscle transfers to improve function and aesthetics, highlighting this method’s potential for similar PBS cases. Moreover, we demonstrated the importance of combining muscle transposition with fascial plication to provide adequate long-term abdominal strength and prevent any abdominal bulging recurrence. Given the limited data available on effective treatment strategies for PBS in adult patients, our procedure aimed to contribute to the development of tailored approaches for this rare disease.

## Case presentation

**Figure 1:**
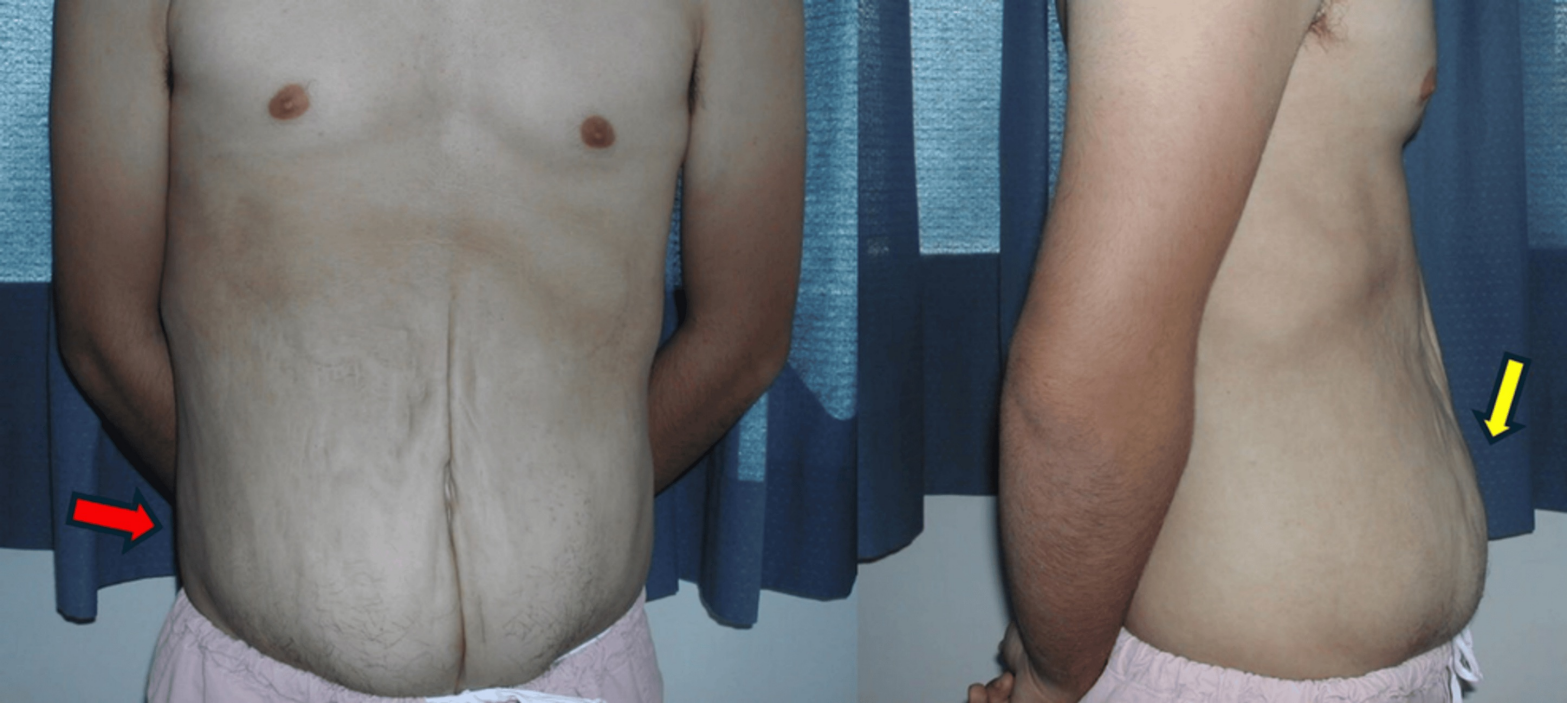
Preoperative view of a 19-year-old patient with prune belly syndrome, showing abdominal flaccidity (red arrow) and distension (yellow arrow)

The study was conducted in accordance with the Declaration of Helsinki. Written informed consent was obtained from the patient for the publication of this case report and the use of the accompanying images and clinical data for publication. This is a case report in which no research has been done. Therefore, it was not necessary that the present case report be evaluated by an ethics committee at our institution. A 19-year-old male patient presented at the Army Recruiting Center to fulfill his military service, and during the mandatory medical examination, he was diagnosed with PBS based on the physical examination, which revealed the presence of abdominal wall flaccidity and distension, as well as on the previously referred operation of distal ureterectomy and vesicoureteral reimplantation (Politano-Leadbetter technique) at the age of two years to correct urinary tract dilation complicated with VUR, and bilateral orchiopexy. Therefore, he was referred to our department for further evaluation and eventual correction of his abdominal wall deformity. Physical examination confirmed the presence of a distended abdomen, associated with hypoplastic anterior abdominal wall musculature, abdominal flaccidity, and the presence of a malformed umbilical remanent (Figure 1). The diagnostic process revealed also the presence of clinically managed chronic renal failure (CRF), scaphoid thorax, and spina bifida.

**Figure 2:**
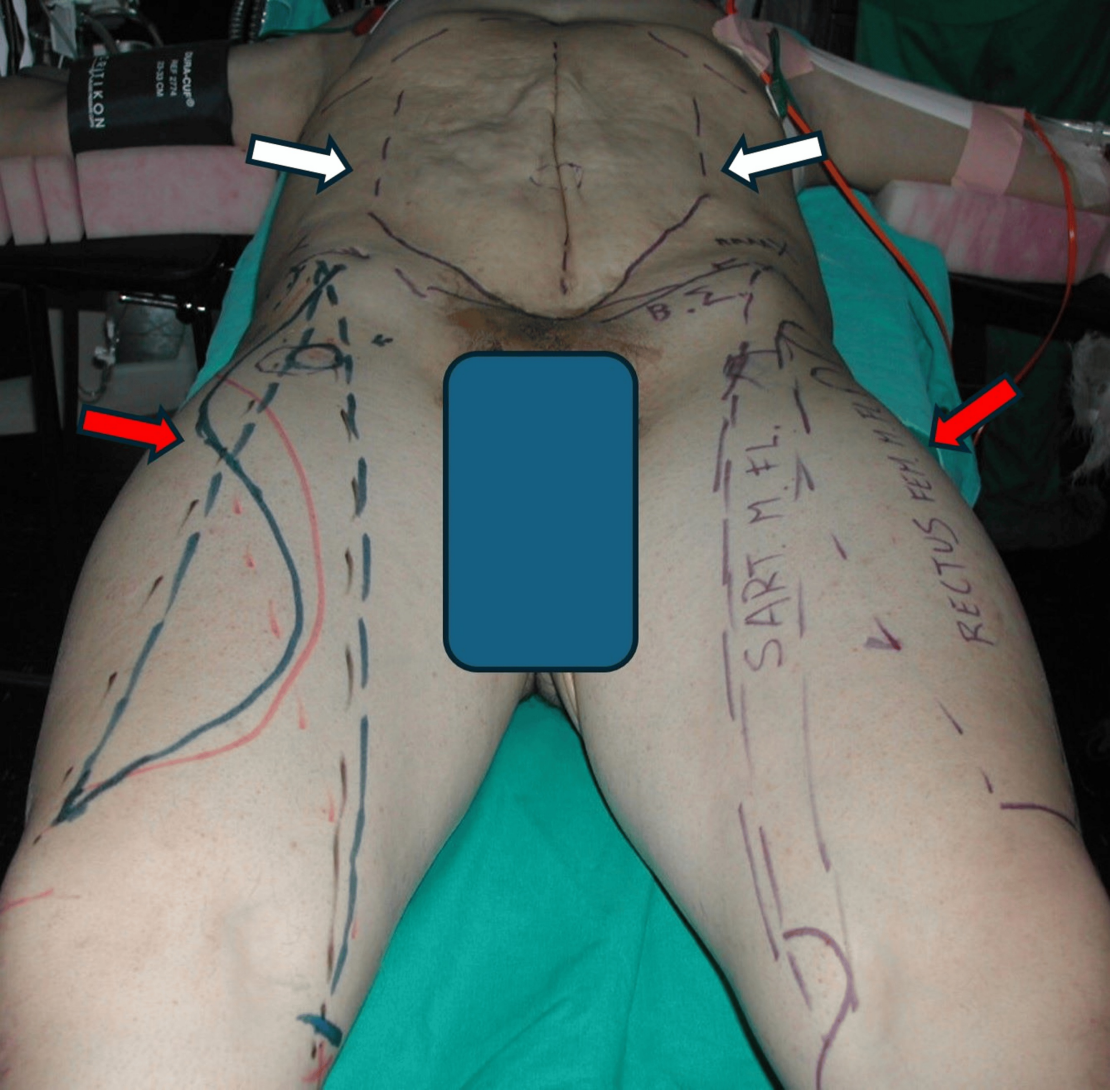
Preoperative markings. White arrows point to the elliptical skin marking, and red arrows point to the design for harvesting the thigh muscles

**Figure 3:**
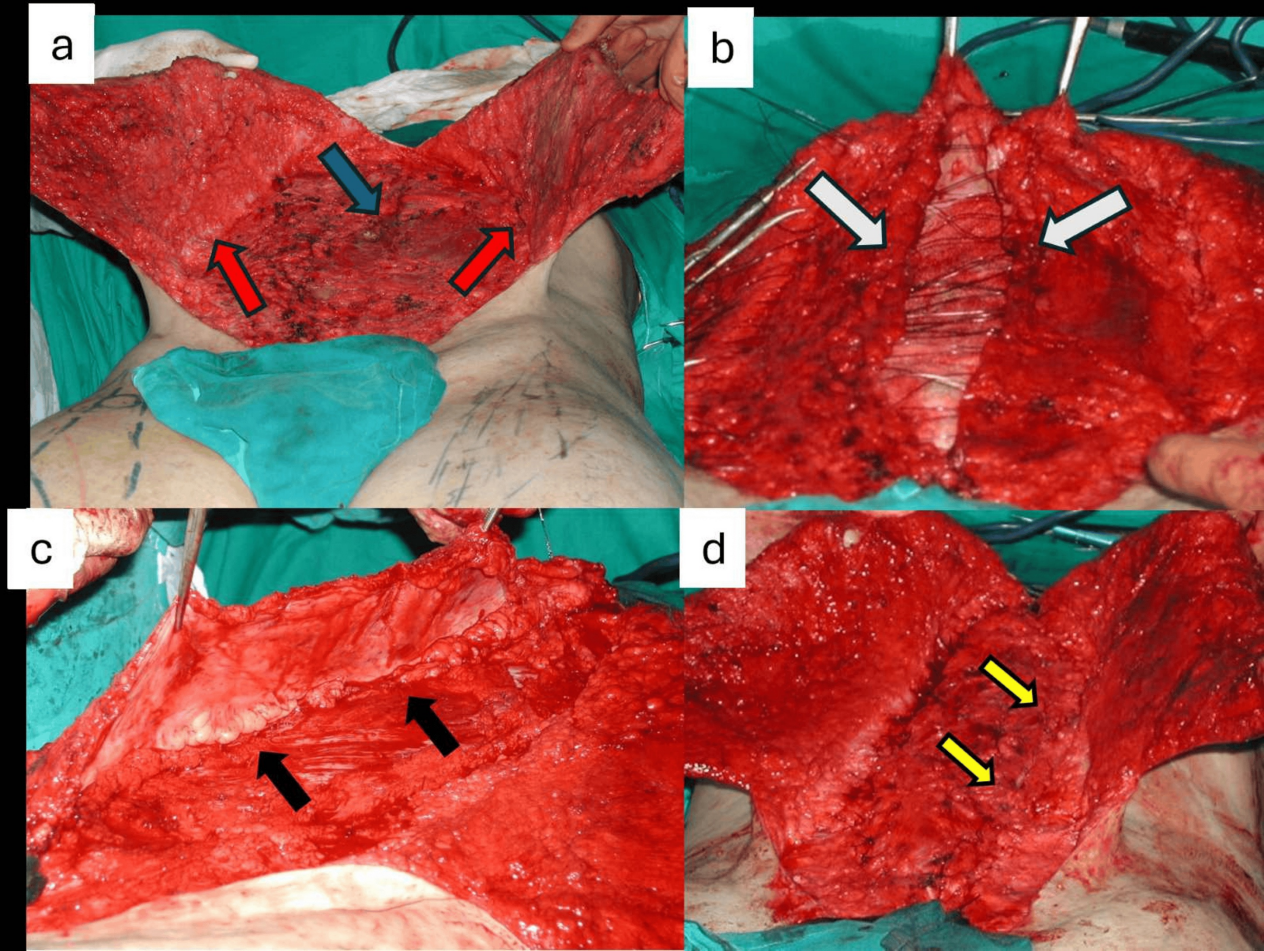
Abdominal skin dissection and fascial plication: (a) extensive lateral dissection of the skin flaps (red arrows), with the presence of hypoplastic abdominal wall musculature (blue arrow). (b) Midline fascial opening and placement of non-absorbable sutures for the fascial plication (white arrows). (c) Advancement and suturing of one fascial flap over the deep surface of another fascial flap (black arrows). (d) Completion of the vest-over-pants fascial plication (yellow arrows)

Based on the severe degree of the abdominal wall musculature deficiency and with the informed patient’s consent, we decided to proceed with a dynamic abdominal wall reconstruction consisting of classic abdominoplasty, fascial plication, and bilateral pedicled rectus femoris and sartorius muscle transfers. Initially, the skin is marked in an elliptical fashion from just below the xiphoid process to just above the pubic area, based on the pinch test for skin redundancy (Figure 2). Then, with the patient in the supine position and under general anesthesia, we proceeded with an inverted-T-shaped incision, extending in the vertical axis at the midline and in the horizontal axis from the anterior superior iliac spine (ASIS) of one side to the opposite side. Skin flaps were dissected laterally off the fascia and extended at the level of the delineated ellipse, and therefore, we confirmed the presence of poorly developed abdominal wall muscles (Figure 3a). The fascia is then opened at the midline, providing access into the peritoneal cavity, and the parietal peritoneum is released from intra-abdominal organs, creating bilateral musculo-aponeurotic fascial flaps (Figure 3b). At this time, one leaf of the fascial flap was transferred across the midline and secured laterally to the deep (peritoneal) side of the contralateral fascial flap with a running Prolene 3/0 suture (Ethicon, Inc., Somerville, US) (Figure 3c). Similarly, the other fascial flap was brought in a vest-over-pants style to the contralateral side and secured to the superficial side of the opposite fascial flap (Figure 3d).

**Figure 4:**
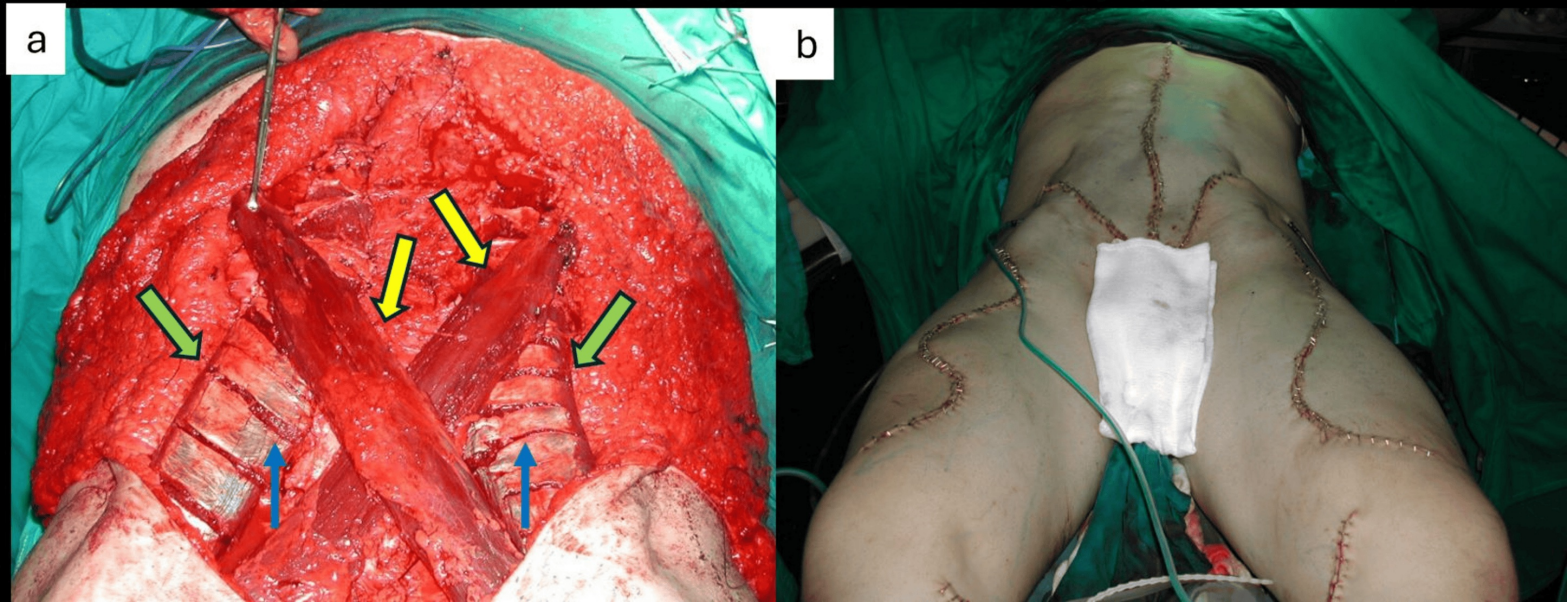
Dynamic abdominal wall reconstruction: (a) transposition of rectus femoris (green arrows) and sartorius (yellow arrows) muscles to the abdomen and fascial scoring of the rectus femoris muscles (blue arrows). (b) Immediate postoperative view

Afterward, bilateral serpiginous incisions were made at the anterior surface of the thighs, and dissection proceeded deeply to identify the rectus femoris and sartorius muscles. Meticulous circumferential dissection was performed around the muscles, preserving their proximal vascular supply and motor innervation. Next, the tendinous insertions of the muscles were cut giving origin to the proximally based pedicled muscular flaps, while in addition, the fascia of rectus femoris muscles was scored to achieve the greater length possible. Then, a subcutaneous tunnel was created through the inguinal region to allow the transposition of the muscles. Consequently, the pedicled muscular flaps were transposed to the reconstructed abdominal wall, where the rectus femoris muscles were secured ipsilaterally and the sartorius muscles in a crossover fashion to the underlying fascia with absorbable sutures (Figure 4a). Transcutaneous drains were placed at the abdominal and thigh regions. Finally, the redundant abdominal skin was excised, and both abdominal and thigh incisions were closed in a layered fashion with absorbable and non-absorbable sutures (Figure 4b).

**Figure 5:**
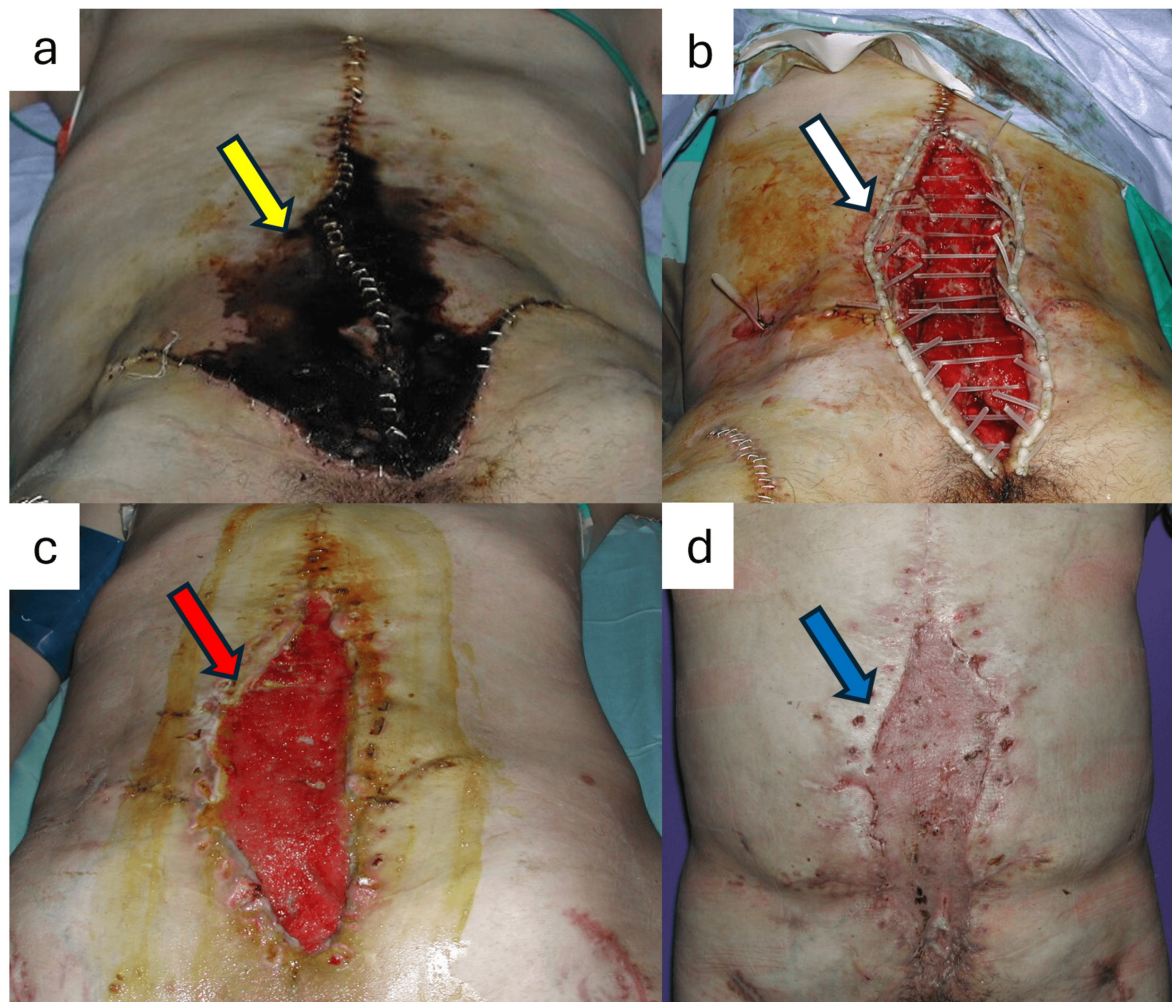
Early postoperative abdominal skin flap necrosis and management: (a) full-thickness abdominal skin flap necrosis 12 days after the abdominal reconstruction (yellow arrow). (b) Debridement of the necrotic tissue and placement of abdominal anchor device (white arrow). (c) Wound bed filled with healthy granulation tissue (red arrow). (d) Coverage of the abdominal defect with split-thickness skin graft (blue arrow)

During the early postoperative period, the patient developed extensive abdominal skin flap necrosis, which was initially treated with surgical debridement and placement of an abdominal anchor device, consisting of elastic silicone bands threaded through the abdominal wall on either side to achieve maximal skin approximation (Figures 5a, 5b). Once the wound bed was adequately filled with healthy granulation tissue, we covered it with the placement of a split-thickness skin graft (Figures 5c, 5d). Three years later, we proceeded to the excision of the grafted area and direct skin closure.

**Figure 6:**
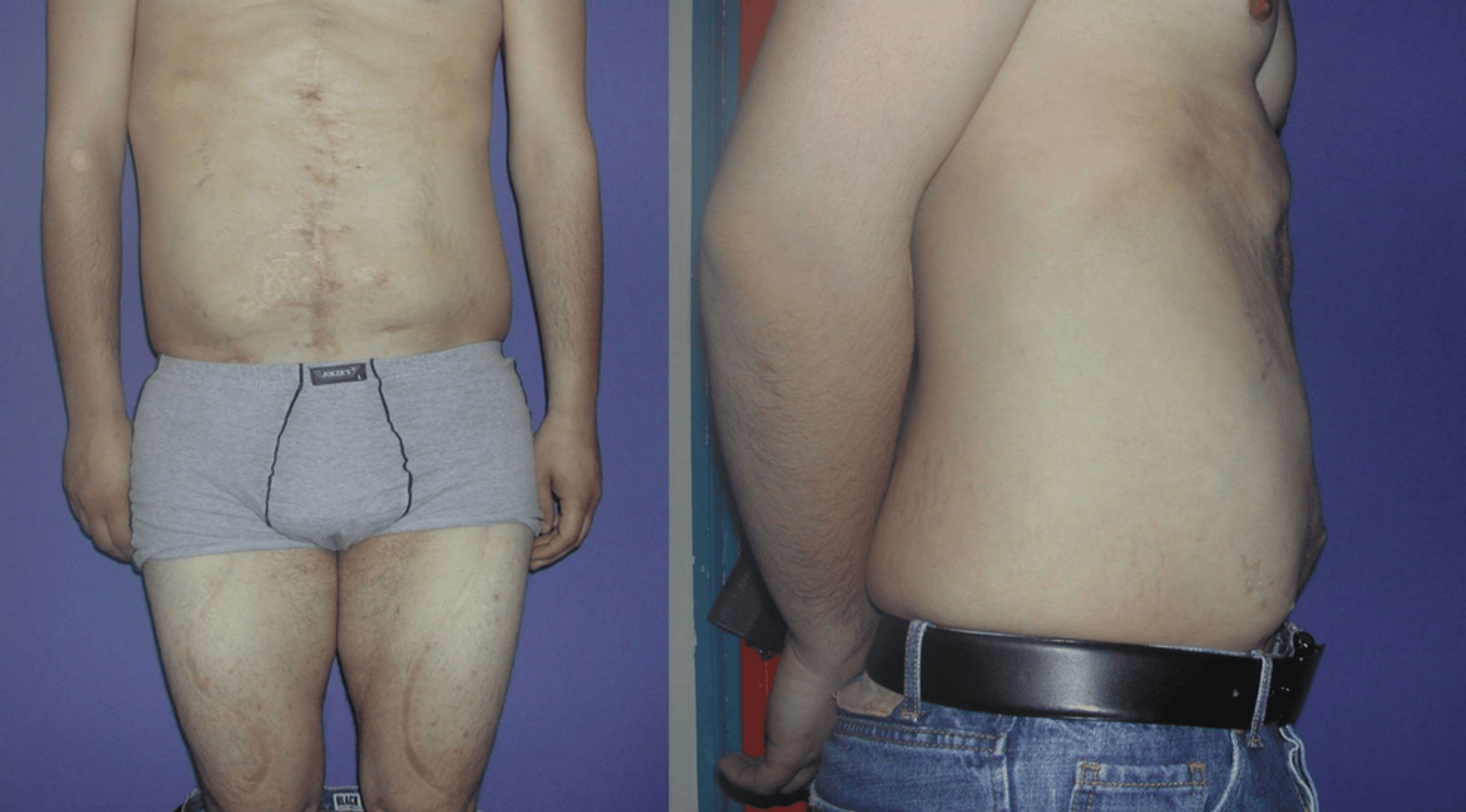
Final postoperative view at the 41-month follow-up with improved aesthetic and functional outcomes

On a follow-up of 41 months from the first operation, the patient achieved significant aesthetic and functional improvement of the abdominal wall, without evidence of recurrent abdominal bulging (Figure 6). Physical examination showed the presence of an active abdominal muscular tonus when the patient was asked to sit up from the supine position. Moreover, there has been no clinically evident impairment of the lower extremity motility, with excellent balance and ambulation.

## Discussion

The timing for abdominal wall reconstruction in PBS depends on the necessity for other interventions and usually is performed during childhood [4,7,8]. Nevertheless, children with moderate abdominal wall musculature deficiency may demonstrate improvement in abdominal flaccidity as they mature, while in other cases, the laxity may worsen during childhood. PBS is rarely seen in the adult population, and most of the published studies are case reports and case series referring mainly to the management of urologic abnormalities [9-11]. To our knowledge, the present is the first case of combined dynamic, with four thigh muscles transferred, and static abdominal wall reconstruction to treat abdominal flaccidity and provide adequate abdominal tonus and strength in an adult with PBS. Different studies used the abdominal fascial plication with or without mesh or acellular dermal matrix interposition to address lateral abdominal bulging in adult patients with PBS, through an open, laparoscopic, and recently robotic approach [12-14]. In our case, we were able to achieve good aesthetic and functional results using autologous tissue instead of a biologic or synthetic material.

The deficiency of the abdominal wall musculature may lead to an ineffective cough and therefore an increase in upper respiratory infections, impaired urination and defecation secondary to reduced Valsalva maneuver, and poor posture and ambulation [6]. Moreover, the muscular defects are not symmetric, and most often, the weakness is more pronounced at the infraumbilical and central part, while the muscles of the upper and lateral abdominal wall are better preserved [6]. Similarly, fascial laxity may be more pronounced in the flank region or focal in a particular abdominal area [15].

Dynamic abdominal wall reconstruction in PBS is a complex procedure with limited bibliographic evidence. The decision to proceed with muscle transfers must be based on the preoperative clinical examination, considering the variability of the different anatomic planes of the anterior abdominal wall, and eventually imaging techniques. The presence of hypoplastic abdominal wall musculature can prevent any major operation, since the existing musculature may strengthen over the years. Moreover, this type of dynamic reconstruction must be performed during later adolescence or adulthood, since at this age the muscles of the thigh gain their full maturity and strength, and therefore, the lower extremity can afford the muscle deficit.

In the present case, we were able to successfully transfer four proximally based pedicled muscle flaps from the thighs to cover as much as possible of the missing abdominal musculature area. All muscles provided an adequate rotation arc. The fascia of the rectus femoris muscles was scored to achieve the greater length possible, while the preservation of the proximal major vascular pedicle of the sartorius muscles permitted complete viability, in accordance with clinical and experimental studies [16,17]. Consequently, with our proposed technique, we achieved “abdominal reanimation,” since the patient improved his ability to move from a sitting to a standing position, as well as the ability to flex and extend his trunk. Similarly, Fearon and Varkarakis reported their experience with combined rectus femoris muscle transposition and fascial plication for PBS patients, with functional transferred muscles, significant improvement of balance and ambulation, and minor abdominal site complications, without recurrent abdominal laxity, and preserved lower extremity motility [18]. Ger and Coryllos reported a satisfactory posture and social activity after an 18-year follow-up of a PBS patient who underwent bilateral rectus femoris muscle transfer [19], while Messing et al. performed bilateral rectus femoris pedicled flaps for detrusor augmentation in PBS [20].

Our patient was in his late adolescence, and the muscles were mature enough to support functional reconstruction of the abdominal wall and the lower extremities to compensate for muscular loss. The lower extremity muscle deficit caused by the transfer of the rectus femoris and sartorius was managed with postoperative physical therapy. Strengthening focused on the iliopsoas to enhance hip flexion and the vastus lateralis, intermedius, and medialis to support knee extension. After these interventions, the patient exhibited a normal gait with no significant abnormalities.

Unfortunately, we experienced abdominal skin flap necrosis due to an extensive lateral dissection, which was reflected also in prolonged hospitalization. We were able to promptly treat this complication and offer an excellent final aesthetic result. Accordingly, Fearon and Varkarakis in a preliminary review reported overall skin/umbilical ischemia of 7% and approximately 20% frequency for reoperations to retighten the abdomen [18], while Lopes et al. described a single comprehensive surgical approach for PBS with 10% frequency for secondary abdominoplasties due to persistent or recurrent flaccidity [8].

Our technique, which combines redundant skin resection, fascial plication (static reconstruction), and muscle transfer (dynamic reconstruction), aims to improve abdominal cosmesis and strength and offer a better quality of life in these complex patients. Already from the early postoperative period, the patient reported improved posture and ambulation, while the long-term follow-up showed restoration of the abdominal wall contour, without evidence of bulging recurrence.

## Conclusions

Our case report demonstrates that anatomic and dynamic reconstruction of the abdominal wall in adult patients with PBS through fascial plication and muscle replacement appears to be a feasible option, able to provide long-term aesthetic and functional improvement. To our knowledge, this is the first case to use four pedicled thigh muscles with motor capabilities and minimal donor site morbidity, able to substitute the missing abdominal musculature in adult PBS patients. We also highlight the challenges in managing adult patients with PBS and the need for further research in this specific population. Detailed preoperative analysis and discussion with the patient can determine the appropriate individualized surgical plan, reducing at the same time the probability of complications. Given the limited literature evidence on PBS management, our case aims to add more bibliographic knowledge on the surgical approach of PBS and therefore improve patient outcomes.
